# Role of goblet cell protein CLCA1 in murine DSS colitis

**DOI:** 10.1186/s12950-016-0113-8

**Published:** 2016-02-04

**Authors:** Nancy A. Erickson, Lars Mundhenk, Samoa Giovannini, Rainer Glauben, Markus M. Heimesaat, Achim D. Gruber

**Affiliations:** Department of Veterinary Pathology, Freie Universität Berlin, Robert-von-Ostertag-Strasse 15, 14163 Berlin, Germany; Medical Department, Division of Gastroenterology, Infectiology and Rheumatology, Charité - Universitätsmedizin Berlin, Hindenburgdamm 30, 12200 Berlin, Germany; Department of Microbiology and Hygiene, Charité - Universitätsmedizin Berlin, Garystrasse 5, 14195 Berlin, Germany

**Keywords:** *Clca1*^*-/-*^, CXCL-1, Keratinocyte chemottractant, CXCL-2, MIP-2α, Inflammation, Signaling molecule, IL-17, E-cadherin, TFF3

## Abstract

**Background:**

The secreted goblet cell protein CLCA1 (chloride channel regulator, calcium-activated-1) is, in addition to its established role in epithelial chloride conductance regulation, thought to act as a multifunctional signaling protein, including cellular differentiation pathways and induction of mucus production. Specifically, CLCA1 has recently been shown to modulate early immune responses by regulation of cytokines. Here, we analyze the role of CLCA1, which is highly expressed and secreted by colon goblet cells, in the course of murine dextran sodium sulfate-induced colitis.

**Findings:**

We compared *Clca1*-deficient and wild type mice under unchallenged and DSS-challenged conditions at various time points, including weight loss, colon weight-length-ratio and histological characterization of inflammation and regeneration. Expression levels of relevant cytokines, trefoil factor 3 and E-cadherin were assessed via quantitative PCR and cytometric bead arrays. Lack of CLCA1 was associated with a more than two-fold increased expression of *Cxcl-1*- and *Il-17*-mRNA during DSS colitis. However, no differences were found between *Clca1*-deficient and wild type mice under unchallenged or DSS-challenged conditions in terms of clinical findings, disease progression, colitis outcome, epithelial defects or regeneration.

**Conclusions:**

CLCA1 is involved in the modulation of cytokine responses in the colon, albeit differently than what had been observed in the lungs. Obviously, the pathways involved depend on the type of challenge, time point or tissue environment.

**Electronic supplementary material:**

The online version of this article (doi:10.1186/s12950-016-0113-8) contains supplementary material, which is available to authorized users.

## Introduction

The goblet cell-derived protein CLCA1 (chloride channel regulator, calcium-activated 1) is thought to act as a multifunctional signaling protein via as yet unidentified molecular pathways. Originally, CLCA1 had been thought to modulate epithelial cell chloride conductance. Subsequent work has shown, however, that it may also induce airway mucus production through an interleukin (IL)-13-mediated cascade [1] and promote spontaneous differentiation while reducing proliferation of Caco-2 cells [2]. In a similar context, CLCA1 expression was proposed as a prognostic factor in colorectal cancer [2, 3].

Recently, CLCA1 was shown to modulate pulmonary cytokine expression in early immune responses, specifically the pro-inflammatory response of human airway macrophages in vitro [4], whereas more complex results have been obtained from mouse models [5, 6]. In *Clca1*-deficient (*Clca1*^*-/-*^) mice, experimental *Staphylococcus (S.) aureus* pneumonia was associated with decreased responses of chemokine (C-X-C motif) ligand (CXCL)-1, a potent neutrophil chemoattractant, with consequently decreased neutrophil recruitment [5]. Furthermore, lack of Clca1 expression yielded reduced responses of the pro-inflammatory cytokine IL-17. In contrast, following intranasal ovalbumin or lipopolysaccharide (LPS) challenge, Clca1-deficiency resulted in increased neutrophil recruitment preceded by CXCL-1 upregulation in the LPS model [6]. Thus, the role of CLCA1 in cytokine modulation seems to be complex and dependent on the stimulus used.

Here, we investigated the proposed function of CLCA1 in modulating the early immune response in the colon, the tissue in which CLCA1 is most highly expressed in man [7] and mice [8]. We chose the dextran sodium sulfate (DSS) challenge model which is commonly used to study early immune reactions in mouse intestine [9]. In a previous DSS colitis study, we failed to observe any effects of Clca1-deficiency on mucus barrier integrity and mucin gene expression [10] which likely would have affected secondary immune responses. Additionally, recent studies have indicated that CLCA1 does not play a role in calcium-activated chloride secretion in the respiratory tract nor does restoration of reduced Clca1 expression rectify the cystic fibrosis electrophysiology defect in the intestine [11, 12]. In light of the absence of such possibly interfering effects, we postulated that possible differences in inflammatory parameters during DSS colitis in the *Clca1*^*-/-*^ model would be due to primary CLCA1 effects on the immune response. We thus compared *Clca1*^-/-^ and wild type (WT) mice under unchallenged and DSS-challenged conditions in terms of key clinical and histopathological parameters as well as expression profiles of select cytokines. Furthermore, we determined expression levels of goblet cell-derived trefoil factor (*Tff*)-3, a key regulator in mucosal repair and protection [13], and E-cadherin (E-cad) which is down-regulated after *Clca1*-knock-down in vitro [3].

## Materials and methods

### Ethics statement, mice and DSS treatment

*Clca1*^-/-^ and WT mice were given 2.5 % DSS for 24 (24 h-group), 48 h (48 h-group) or for 7 days with 2 consecutive days of water (7 d-group) as described [10]. For ethics statement, selection and treatment see Additional file 1.

### Weight loss, colon weight-length-ratio and sampling

In the 7 d-group, weight loss was determined in the course of DSS administration and colon weight-length-ratio at necropsy. Colons of all groups were equally sectioned from proximal to distal for histopathology which were immediately fixed in 4 % buffered formalin. For Reverse Transcriptase-quantitative PCR (RT-qPCR) and organ culture, sections were opened longitudinally, flushed with ice-cold Dulbecco’s phosphate buffered saline (biowest, Nuaille, France) and either snap frozen in liquid nitrogen and stored at – 80 °C or immediately processed, respectively.

### Histopathology

4 μm thick formalin-fixed, paraffin-embedded, hematoxylin and eosin-stained sections of proximal and distal colon were evaluated separately by veterinary pathologists in a blinded fashion according to a scoring scheme (Additional file 2).

### RNA isolation and reverse transcriptase-qPCR

Total RNA isolation, primer and probe design, RT-qPCR and data analysis were performed as described [5, 10]. Transcript expression levels of *Cxcl-1*, *Cxcl-2*, *Il-17*, *Tnf*, *Ifnγ*, *Tff3* and *E-cad* were determined and normalized to the internal reference genes glyceraldehyde-3-phosphate dehydrogenase (*Gapdh*), elongation factor-1α (*Ef-1α*) and ß-2 microglobulin (*B2m*) as described [5]. Primers and probes are listed in Additional file 3.

### Cytometric bead array

Colon cultures were established and supernatants processed as described [14] and stored at -80 °C until further analysis. Cytokine concentrations of CXCL-1, monocyte chemoattractant protein (MCP)-1, TNF, IFNγ, IL-1β, -2, -6, -13 and -17A were determined via cytometric bead array using a FACSCantoII and the FacsDiva software (all BD Biosciences, Heidelberg, Germany) as described [15, 16].

### Statistics

Statistical analyses via Mann–Whitney-U test and graphical illustrations were performed using GraphPad PRISM 6 (GraphPad Software Inc., La Jolla, USA) and data are expressed as mean ± standard error of the mean (SEM) except for RT-qPCR data. Here, data are expressed as single value fold change of which a more than two-fold difference between the genotypes was considered relevant.

## Results and discussion

**Fig. 1:**
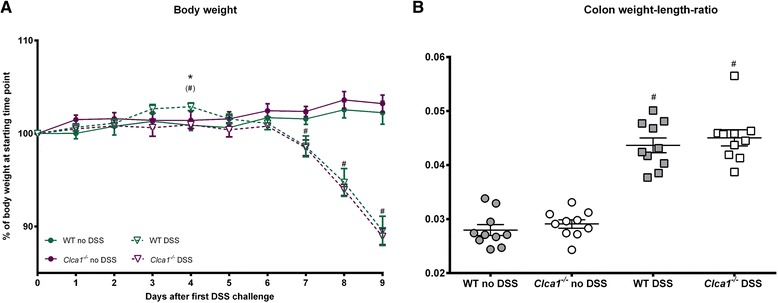
Similar body weights and colon weight-length-ratios of *Clca1* ^*-/-*^ and WT mice. **a** From day 7 on, both DSS-treated genotypes showed a continuous weight loss to 88.9 ± 0.9 % and 89.5 ± 1.6 % at day 9 whereas the unchallenged mice continued a slight overall weight gain to 103.2 ± 0.9 % and 102.2 ± 1.2 % for *Clca1* ^*-/-*^ and WT mice, respectively. At day 4, DSS-treated WT mice had higher body weights than the WT controls and the DSS-challenged *Clca1* ^*-/-*^ group, the relevance of which, however, remains questionable. *n* = 10 – 30 per group. **b** The colon weight-length-ratios increased during DSS colitis compared to unchallenged controls from 0.027 ± 0.001 and 0.027 ± 0.001 to 0.044 ± 0.002 and 0.043 ± 0.001 for *Clca1* ^*-/-*^ and WT, respectively, however, with no difference between the genotypes. *n* = 10 per group. ^#^ *p* < 0.05 vs. the unchallenged control group. *p < 0.01 between the genotypes

The percentile body weights declined following day 6 of DSS application (Fig. 1a) and colon weight-length-ratios increased (Fig. 1b), without any difference between the genotypes (Fig. 1) which is in line with the genotype-independent decrease of stool consistency and increase of fecal blood content in the DSS colitis model described earlier [10]. At day 4, DSS-treated WT mice had higher body weights compared to the DSS-challenged *Clca1*^*-/-*^ mice but also to unchallenged WT controls. In this context and being only 2 % in difference between the genotypes, this single statistically significant data point is biologically questionable. Alternatively, in the context of *Ifnγ* mRNA decrease at early time points (see below) in *Clca1*^*-/-*^ mice, this may indicate changes in metabolism in *Clca1*-deficient mice only. Importantly, Clca1-deficiency had no impact on any clinical parameter tested which is in line with the previous respiratory challenge model [5].

**Fig. 2:**
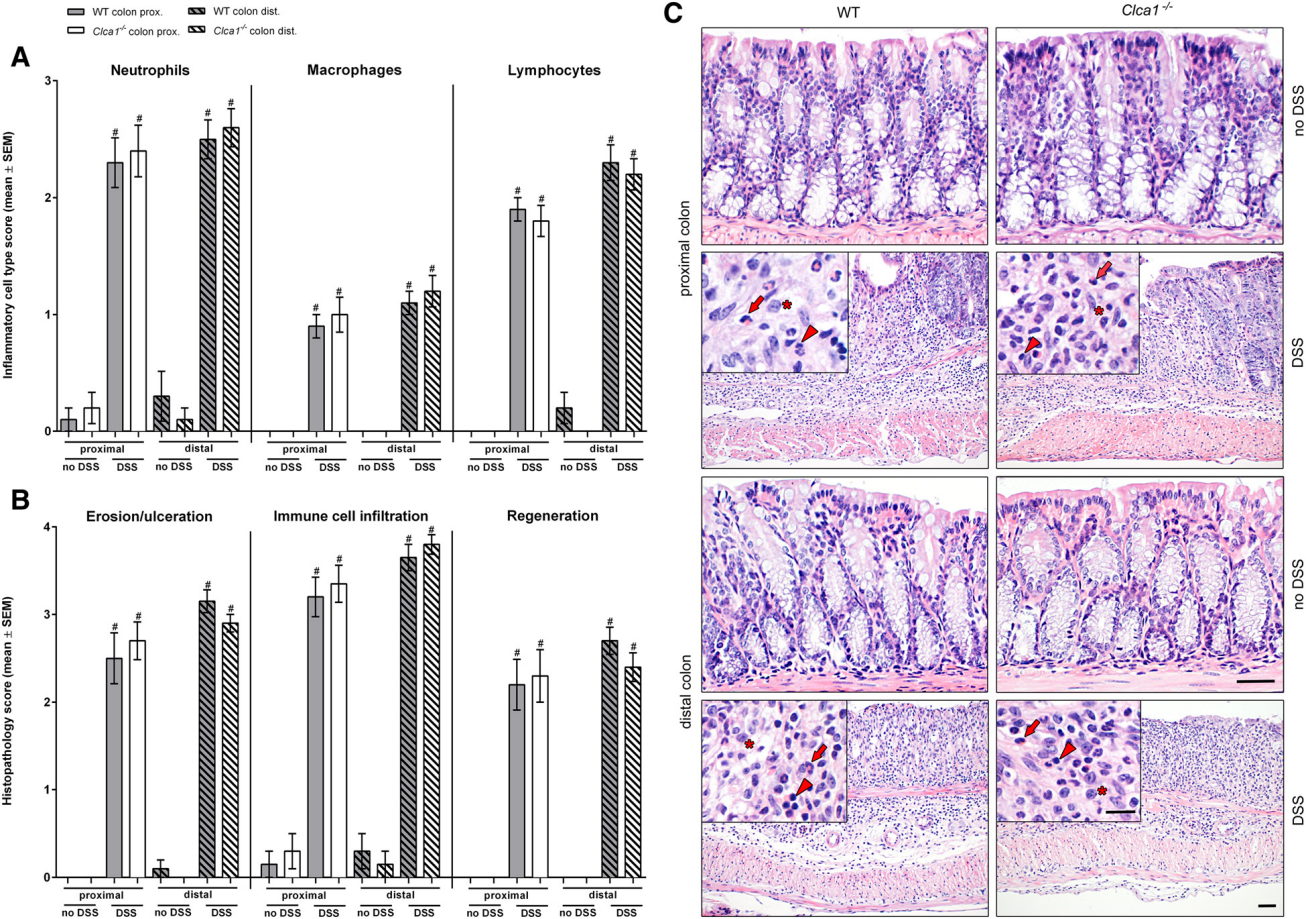
Similar inflammation and regeneration in *Clca1* ^*-/-*^ and WT mice during DSS colitis. During DSS colitis, the **a** abundance of neutrophils, macrophages and lymphocytes, **b** the histopathology scores for erosion/ulceration and immune cell infiltration increased as well as regeneration in proximal and distal colon following a 7-day DSS colitis induction and administration of water for 2 consecutive days. No differences were observed between *Clca1* ^*-/-*^ and WT mice. **c** Representative histopathology of proximal and distal colon of DSS-treated *Clca1* ^*-/-*^ and WT mice and water controls; neutrophils (arrows), macrophages (asterisks) and lymphocytes (arrowheads). Bars: 20 μm (no DSS and insets) and 50 μm (DSS). *n* = 10 per group. ^#^ *p* < 0.05 vs. the unchallenged control group

Histopathologically, Clca1-deficiency did not result in any effect on the extent and nature of inflammatory cells, epithelial defects and regeneration. During DSS colitis, neutrophils, macrophages and lymphocytes (Fig. 2a and c, insets) as well as erosion/ulceration, immune cell infiltration and regeneration (Fig. 2b and c) increased in proximal and distal colon, clearly reflecting the expected inflammation. At 24 und 48 h, no significant immune cell infiltrations or histopathologic alterations were observed (Additional file 4). In contrast to previous studies on airway inflammation in which neutrophil responses were either decreased in *Clca1*^*-/-*^ mice after *S. aureus* infection [5] or increased after LPS challenge [6], no difference was observed histopathologically in the colitis model between the genotypes.

**Fig. 3:**
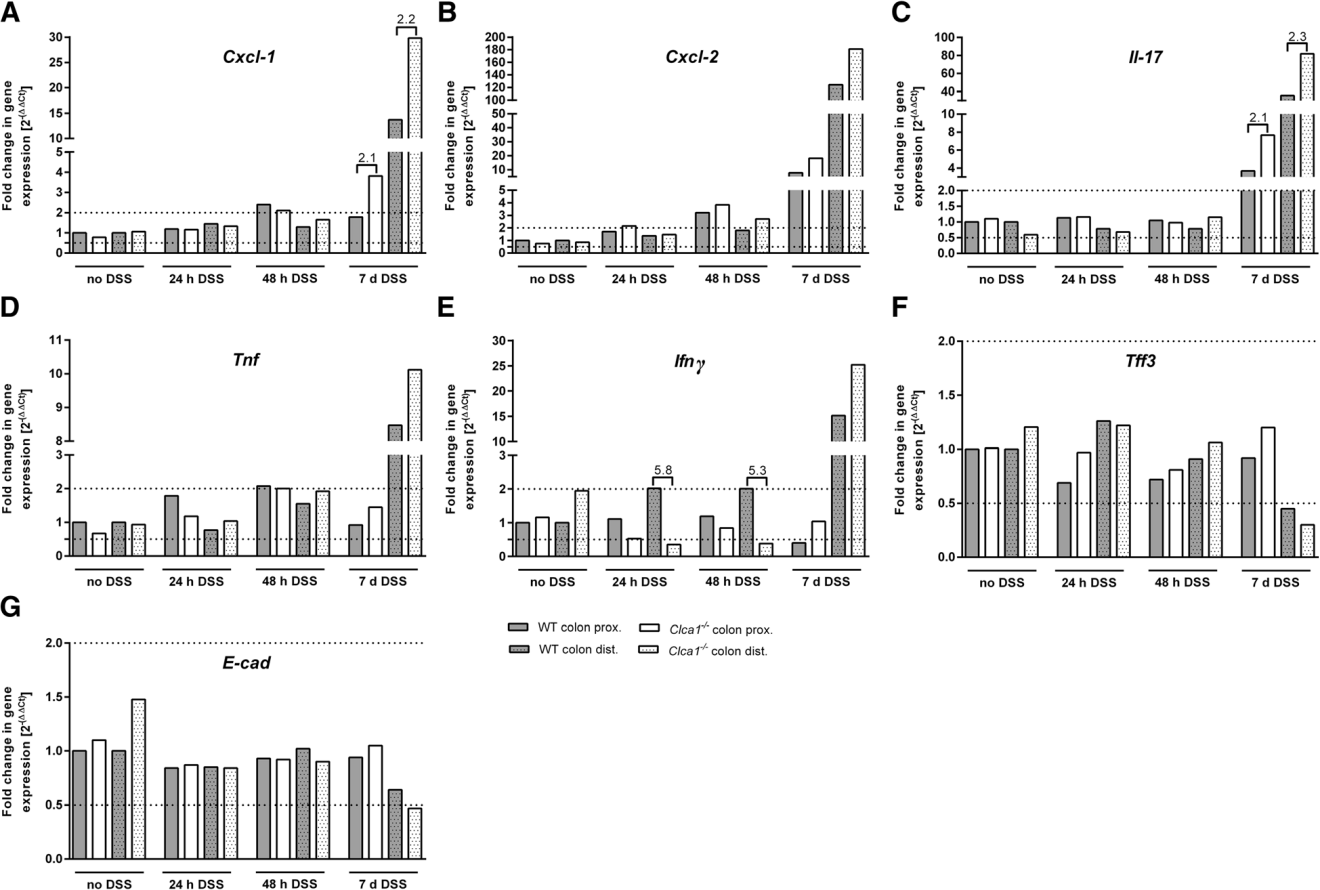
Increase in *Cxcl-1*- and *Il-17*-mRNA expression in *Clca1* ^*-/-*^ mice during DSS-challenge. During DSS colitis, **a** *Cxcl-1*, **b** *Cxcl-2,* **c** *Il-17,* **d** *Tnf* and **e** *Ifnγ* were upregulated in the distal colon as the prime target site of DSS and sporadically also in the proximal colon. However, only *Cxcl-1* and *Il-17* showed a greater than two-fold increase of mRNA in the distal colon of *Clca1* ^*-/-*^ compared to WT. At earlier time points, *Cxcl-2* was upregulated at 24 h in *Clca1* ^*-/-*^ proximal colon and at 48 h in all except for WT distal colon. At 48 h, *Cxcl-1, Tnf* and *Ifnγ* were upregulated in proximal colon of *Clca1* ^*-/-*^ and WT, in proximal and in distal colon of WT mice, respectively. *Ifnγ* showed a more than five-fold decrease in the distal colon of DSS-challenged *Clca1* ^*-/-*^ compared to WT at 24 and also at 48 h. Expression of **f** *Tff3* in distal *Clca1* ^*-/-*^ and WT colon and of **g** *E-cad* in distal *Clca1* ^*-/-*^ colon was lower during colitis conditions, however, without difference between the genotypes. Dotted lines indicate a fold change of 0.5 and 2, respectively, as limits for valid statement of lowered or elevated expressions. A greater than two-fold difference in fold change was considered as relevant difference between the genotypes. Ct, cycle threshold. Relative quantification and comparison of groups were performed by the ∆ΔCt method using unchallenged WT animals as controls (fold change = 1). *n* = 9–18 per group

As expected, mRNA expressions of *Cxcl-1*, *Cxcl-2, Il-17, Tnf* and *Ifnγ* (Fig. 3a to e) were overall upregulated in distal and, sporadically, also in proximal colon during DSS colitis. These cytokines, except for *Il-17*, occasionally were slightly elevated at earlier time points.

However, *Cxcl-1* and *Il-17* (Fig. 3a and c) showed a greater than two-fold increase of mRNA copy numbers in distal colon of *Clca1*^*-/-*^ compared to WT mice during colitis. The significance of the transiently lower *Ifnγ* expression levels in the distal colon of DSS-challenged *Clca1*^*-/-*^ compared to WT at 24 and 48 h (Fig. 3e) is unclear due to lack of histologically evident immune cell infiltration at these time points (Additional file 4). Expression of *Tff3* mRNA in distal colon of *Clca1*^*-/-*^ and WT mice and of *E-cad* in distal *Clca1*^*-/-*^ colon (Fig. 3f and g) was lower during colitis, likely due to destruction of goblet and epithelial cells, respectively, without differences between the genotypes.

**Fig. 4:**
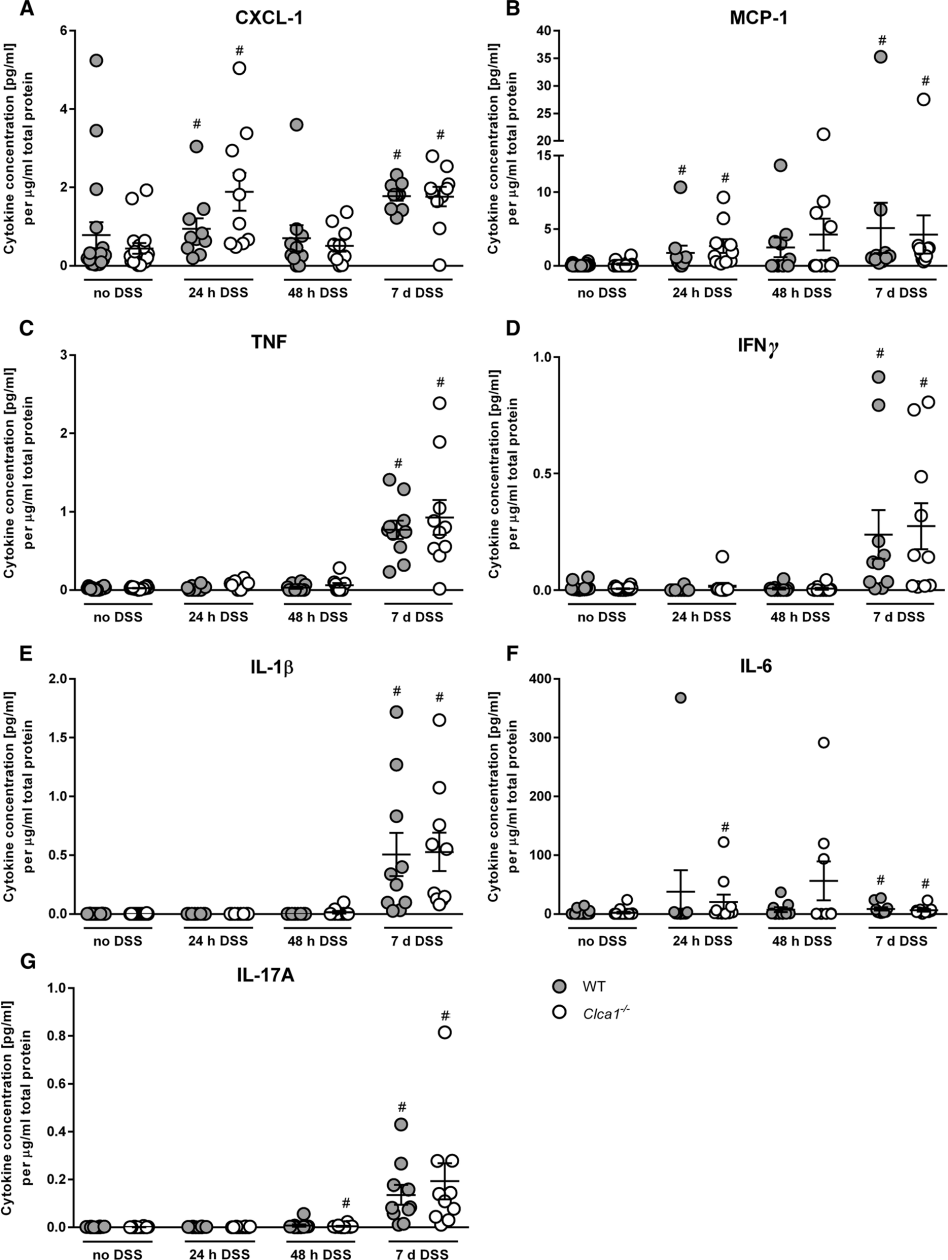
Inflammatory cytokines are similarly upregulated on protein level in DSS colitis between the genotypes. Cytokines were determined on the protein level in colon supernatant via cytometric bead array and normalized to the respective total protein concentration. **a** CXCL-1, **b** MCP-1, **c** TNF, **d** IFNγ, **e** IL-1β, **f** IL-6 and **g** IL-17A were elevated during DSS colitis without any difference between the genotypes. CXCL-1, MCP-1 and IL-6, the latter only in *Clca1* ^-/-^, seemed also slightly elevated at 24 h as well as IL-17A in *Clca1* ^-/-^ at 48 h after DSS challenge, however, without statistical significance between the genotypes. ^#^ *p* < 0.05 vs. the unchallenged control group. *n* = 9–18 per group. Only if more than 5 mice had detectable protein values, the group was considered for valid statement of significance

CXCL-1, MCP-1, TNF, IFNγ, IL-1β, -6 and -17A proteins (Fig. 4a to g) were similarly elevated during DSS colitis, CXCL-1 and MCP-1 also slightly at 24 h of DSS-challenge in both genotypes, IL-6 at 24 and IL-17A at 48 h in the *Clca1*^*-/-*^ mice.

The cytokines IL-2 and -13 were below the detection limits at all time points.

Interestingly, differential expression of *Cxcl-1* mRNA has also been found in respiratory Clca1-deficient mouse models [5], consequentially with differences in CXCL-1 protein level and neutrophil recruitment [5, 6]. Additionally, *Il-17* was also differentially expressed in one model [5]. The prominently higher mRNA expression levels of *Cxcl-1* and *Il-17* in *Clca1*^*-/-*^ DSS colitis mice may point towards initial regulatory events. This may become obvious on protein level at later time points as had been seen in the *S. aureus* pneumonia model [5]. The increase (1.4 fold) of IL-17A in the *Clca1*^*-/-*^ mice of the 7-d group may confirm this notion.

Despite being opposite to the *S. aureus* pneumonia model in which *Cxcl-1* and *Il-17* were decreased in *Clca1*^*-/-*^ mice [5], the DSS colitis data are in line with increased CXCL-1 responses following respiratory LPS-challenge [6]. Early Clca1-linked immune response modulation therefore seems to depend on the stimulus used. As solely LPS-mediated Toll-like receptor 4 signaling seems to be important for neutrophil recruitment, control of bacterial translocation and epithelial repair in acute DSS colitis [17], this model possibly shares similarities with the respiratory LPS challenge.

Our findings confirm the link of CLCA1 to early immune response modulation with a specific effect on *Cxcl-1* and *Il-17* albeit not decisive for clinical outcome. Potentially overlapping effects of CLCA1, including anion conductance and cellular differentiation, will have to be taken into account when further deciphering the interaction of Clca1 with *Cxcl-1* and *Il-17* in early immune responses.

## Additional files

Additional file 1:
**Ethics statement, selection and treatment.** (PDF 34 kb) Additional file 2:
**Histopathological scoring scheme.** (PDF 80 kb) Additional file 3:
**Quantitative RT-PCR – primer and probe sequences.** (PDF 108 kb) Additional file 4:
**Results of histopathology scoring of 24 and 48 h-groups.** (PDF 81 kb)

## Abbreviations

CLCA: Chloride channel regulator, calcium-activated
gob-5: Goblet cell protein-5
DSS: Dextran sodium sulfate
*Clca1*^*-/-*^: *Clca1*-deficient
Cxcl: Chemokine (C-X-C motif) ligand
IL: Interleukin
LPS: Lipopolysaccharide
WT: Wild type
RT-qPCR: Reverse transcriptase quantitative polymerase chain reaction
Tff: Trefoil factor
IFNγ: Interferone gamma
FELASA: Federation of Laboratory Animal Science Associations
h: Hours
d: Days
mRNA: Messenger ribonucleic acid
TNF: Tumor necrosis factor
Gapdh: Glyceraldehyde-3-phosphate dehydrogenase
Ef-1α: Elongation factor 1alpha
B2m: beta-2 microglobulin
MCP: Monocyte chemoattractant protein
SEM: Standard error of the mean
pg: Picograms
μg: Micrograms
ml: Milliliters
*S. aureus*: *Staphylococcus aureus*
KC: Keratinocyte chemoattractant
vs.: Versus
MIP-2α: Macrophage inflammatory protein 2-alpha
E-cad: E-cadherin (alias Cdh1)
