# Supplementary material for: Role of goblet cell protein CLCA1 in murine DSS colitis
Source: J Inflamm (Lond). 2016 Feb 4;13:5. doi: 10.1186/s12950-016-0113-8 (PMC4743195; doi:10.1186/s12950-016-0113-8)
Supplement: Additional file 1: — Ethics statement, selection and treatment. (PDF 34 kb) [file 12950_2016_113_MOESM1_ESM.pdf]

Additional file 1: Ethics statement, selection and treatment

All animal procedures were conducted at the ‘‘Research Institutes for Experimental Medicine”, Charité - Universitätsmedizin Berlin, Germany, in accordance with the FELASA guidelines [1] and approved by the local governmental authorities (State Office of Health and Social Affairs Berlin, approval IDs: T 0394/12; G 0170/12).

Weight- and age-matched female *Clca1*$^{-/-}$ and WT mice were given 2.5 % DSS (MW 36,000 - 50,000, 17.1 % sulfur substitution, no detectable free sulfate, pH 7.1, MP Biomedicals, LLC., Illkirch, France) for 24 (24 h-group) or 48 hours (48 h-group) to allow for the investigation of initial DSS effects, and for 7 days with 2 consecutive days of tap water only (7 d-group) for monitoring effects during acute colitis with beginning regenerative effects as described [2].

Solely female mice were used in the experiment due to ethical and animal welfare reasons. Using female mice reduces the risk of stress and rank fights with subsequent injuries and the possibility of stress-induced weight loss. Second, as females are more resistant to DSS compared to male mice [3], the risk of the animals losing weight too rapidly within the experiment is reduced. A too rapid weight loss could have lead to an exclusion of animals from the experiment in terms of humane end points which would have increased the overall number of animals used in the experiment.

References:

1. Mahler M, Berard M, Feinstein R, Gallagher A, Illgen-Wilcke B, Pritchett-Corning K, Raspa M, Revision FWG: **FELASA recommendations for the health monitoring of mouse, rat, hamster, guinea pig and rabbit colonies in breeding and experimental units.** Laboratory Animals 2014, **48**:178-192.

2. Erickson NA, Nystrom EE, Mundhenk L, Arike L, Glauben R, Heimesaat MM, Fischer A, Bereswill S, Birchenough GM, Gruber AD, Johansson ME: **The Goblet Cell Protein Clca1**

(Alias mClca3 or Gob-5) Is Not Required for Intestinal Mucus Synthesis, Structure and Barrier Function in Naive or DSS-Challenged Mice. PLoS One 2015, **10**:e0131991.

3. Mahler M, Bristol IJ, Leiter EH, Workman AE, Birkenmeier EH, Elson CO, Sundberg JP: **Differential susceptibility of inbred mouse strains to dextran sulfate sodium-induced colitis.** American Journal of Physiology - Gastrointestinal and Liver Physiology 1998, **274**: G544-G551
