# Supplementary material for: Role of goblet cell protein CLCA1 in murine DSS colitis
Source: J Inflamm (Lond). 2016 Feb 4;13:5. doi: 10.1186/s12950-016-0113-8 (PMC4743195; doi:10.1186/s12950-016-0113-8)
Supplement: Additional file 2: — Histopathological scoring scheme. (PDF 80 kb) [file 12950_2016_113_MOESM2_ESM.pdf]

Additional file 2: Histopathological scoring scheme*

**Cell type score for neutrophils, macrophages or lymphocytes**	
0	Absent
1	Few
2	Moderate
3	Abundant

**Erosion/ulceration**			
Depth of erosions/ulcerations		Extent of erosions/ulcerations	
0	None	0	None (0)
1	*Lamina epithelialis*	1	Punctate (1)
2	+ *Lamina propria mucosae*	2	Minimal (2)
3	+ *Lamina muscularis propriae*	3	Moderate (3)
4	+ *Tela submucosa*	4	Widespread (> 3)

**Immune cell infiltration**			
Presence of immune cell infiltration		Extent of immune cell infiltration	
0	None	0	None
1	Minimal	1	Mucosal
2	Mild	2	Mucosal + submucosal
3	Moderate	3	Mucosal + submucosal + muscle
4	Severe	4	Mucosal + submucosal + muscle + serosa

**Regeneration**	
0	No reepithelization
1	Hyperplasia of crypt epithelium
2	Early reepithelization
3	Partial reepithelization
4	Complete reepithelization

*modified from Rachmilewitz D, Karmeli F, Takabayashi K, Hayashi T, Leider-Trejo L, Lee J, Leoni LM, Raz E: **Immunostimulatory DNA ameliorates experimental and spontaneous murine colitis.** *Gastroenterology* 2002, **122:**1428-1441.
