# Supplementary material for: Role of goblet cell protein CLCA1 in murine DSS colitis
Source: J Inflamm (Lond). 2016 Feb 4;13:5. doi: 10.1186/s12950-016-0113-8 (PMC4743195; doi:10.1186/s12950-016-0113-8)
Supplement: Additional file 3: — Quantitative RT-PCR – primer and probe sequences. (PDF 108 kb) [file 12950_2016_113_MOESM3_ESM.pdf]

Additional file 3: Quantitative RT-PCR – primer and probe sequences

Gene	Genbank Accession no.	Oligonucleotide Sequences (5’-3’)	Reference
Cxcl1(KC)	NM_008176.3	Primer (upstream) GATGCTAAAAGGTGTCCCCA<br>Primer (downstream)AGACTGCTCTGATGGCACCT<br>TaqMan Probe FAM-GTCAGAAGCCAGCGTTCAC-BHQ	*
Cxcl2(MIP-2α)	NM_009140.2	Primer (upstream) CTGAACAAAGGCAAGGCTAACT<br>Primer (downstream)ACCTGGAAAGGAGGAGCCT<br>TaqMan Probe FAM-CTTTGGTTCTTCCGTTGAGG-BHQ	*
Il-17	NM_010552	Primer (upstream) GCTCCAGAAGGCCCTCAGA<br>Primer (downstream)CTCTCCACCGCAATGAAGACCCTGA<br>TaqMan Probe FAM-AGCTTTCCCTCCGCATTGA-BHQ	[1]
TNF	NM_013693.3	Primer (upstream) CATCTTCTCAAAATTCGAGTGACAA<br>Primer (downstream)TGGGAGTAGACAAGGTACAACCC<br>TaqMan Probe FAM-CACGTCGTAGCAAACCACCAAGTGGA-BHQ*	[2]
INFγ	NM_008337.3	Primer (upstream) GAACTGGCAAAAGGATGGTGAC<br>Primer (downstream)GTTGCTGATGGCCTGATTGTC<br>TaqMan Probe FAM-TGAAAATCCTGCAGAGCCAGA-BHQ*	ID 8139 [3]
Tff3	NM_011575.2	Primer (upstream) TAATGCTGTTGGTGGTCCTG<br>Primer (downstream)CAGCCACGGTTGTTACACTG<br>TaqMan Probe FAM-ATGTCAGAGTGGACTGTGGCTAC-BHQ	*
E-Cad (Cdh1)	NM_009864.2	Primer (upstream) GGCTTCAGTTCCGAGGTCTA<br>Primer (downstream)AGTCTTCCGAAAAGAAGGCTG<br>TaqMan Probe FAM-TCCTGGGCAGAGTGAGATTT -BHQ	*
Ef-1a	NM_010106.2	Primer (upstream) AAAAACGACCCACCAATGG<br>Primer (downstream)GGCCTGGATGGTTCAGGATA<br>TaqMan Probe FAM- AGCAGCTGGCTTCACGCTCAGGTG -BHQ	[4]
B2m	NM_009735.3	Primer (upstream) ATTCACCCCCACTGAGACTGA<br>Primer (downstream)CTCGATCCCAGTAGACGGTC<br>TaqMan Probe FAM-TGCAGAGTTAAGCATGACAGTATGGCCG -BHQ	[5]
Gapdh	NM_008084.2	Primer (upstream) TCACCACCATGGAGAAGG<br>Primer (downstream)GCTAAGCAGTTGGTGGTGCA<br>TaqMan Probe FAM-ATGCCCCCATGTTTGTGATGGGTGT-BHQ	[1]

*Primers and/or probes designed by authors.

References

1. Giulietti A, Overbergh L, Valckx D, Decallonne B, Bouillon R, Mathieu C: **An overview of real-time quantitative PCR: applications to quantify cytokine gene expression.** *Methods* 2001, **25:**386-401.
2. Innamorato NG, Rojo AI, Garcia-Yague AJ, Yamamoto M, de Ceballos ML, Cuadrado A: **The transcription factor Nrf2 is a therapeutic target against brain inflammation.** *J Immunol* 2008, **181:**680-689.
3. Pattyn F, Robbrecht P, De Paepe A, Speleman F, Vandesompele J: **RTPrimerDB: the real-time PCR primer and probe database, major update 2006.** *Nucleic Acids Res* 2006, **34:**D684-688.
4. Braun J, Mundhenk L, Range F, Gruber AD: **Quantitative expression analyses of candidates for alternative anion conductance in cystic fibrosis mouse models.** *J Cyst Fibros* 2010, **9:**351-364.
5. Norris MD, Burkhart CA, Marshall GM, Weiss WA, Haber M: **Expression of N-myc and MRP genes and their relationship to N-myc gene dosage and tumor formation in a murine neuroblastoma model.** *Med Pediatr Oncol* 2000, **35:**585-589.
