# Supplementary material for: Role of goblet cell protein CLCA1 in murine DSS colitis
Source: J Inflamm (Lond). 2016 Feb 4;13:5. doi: 10.1186/s12950-016-0113-8 (PMC4743195; doi:10.1186/s12950-016-0113-8)
Supplement: Additional file 4: — Results of histopathology scoring of 24 and 48 h-groups. (PDF 81 kb) [file 12950_2016_113_MOESM4_ESM.pdf]

Additional file 4: Results of histopathology scoring of 24 and 48 h-groups

	Clca1$^{-/-}$ 24 h DSS proximal colon										*Clca1*$^{-/-}$ 24 h DSS distal colon									
	1	2	3	4	5	6	7	8	9	10	1	2	3	4	5	6	7	8	9	10
Cell type score neutrophils	0	0	1	0	0	0	0	0	0	0	0	0	0	0	0	0	0	0	0	0
Cell type score macrophages	0	0	0	0	1	0	0	0	0	0	0	0	0	0	0	0	0	0	0	0
Cell type score lymphocytes	0	0	0	1	2	0	0	0	0	0	0	0	0	0	0	0	0	0	0	0
Erosion/ulceration - depth	0	0	0	0	0	0	0	0	0	0	0	0	0	0	0	0	0	0	0	0
Erosion/ulceration - extent	0	0	0	0	0	0	0	0	0	0	0	0	0	0	0	0	0	0	0	0
Immune cell infiltration - presence	0	0	0	0	0	0	0	0	0	0	0	0	0	0	0	0	0	0	0	0
Immune cell infiltration - extent	0	0	0	0	0	0	0	0	0	0	0	0	0	0	0	0	0	0	0	0
Regeneration	0	0	0	0	0	0	0	0	0	0	0	0	0	0	0	0	0	0	0	0

	WT 24 h DSS proximal colon										WT 24 h DSS distal colon									
	1	2	3	4	5	6	7	8	9	10	1	2	3	4	5	6	7	8	9	10
Cell type score neutrophils	0	0	0	0	0	0	0	0	0	0	0	0	0	0	0	0	0	0	0	0
Cell type score macrophages	0	0	0	0	0	0	0	0	1	0	0	0	0	0	0	0	0	0	0	0
Cell type score lymphocytes	0	0	0	1	0	0	0	1	1	1	0	0	0	1	1	0	0	0	0	1
Erosion/ulceration - depth	0	0	0	0	0	0	0	0	0	0	0	0	0	0	0	0	0	0	0	0
Erosion/ulceration - extent	0	0	0	0	0	0	0	0	0	0	0	0	0	0	0	0	0	0	0	0
Immune cell infiltration - presence	0	0	0	0	0	0	0	0	0	0	0	0	0	0	0	0	0	0	0	0
Immune cell infiltration - extent	0	0	0	0	0	0	0	0	0	0	0	0	0	0	0	0	0	0	0	0
Regeneration	0	0	0	0	0	0	0	0	0	0	0	0	0	0	0	0	0	0	0	0

	Clca1$^{-/-}$ 48 h DSS proximal colon										*Clca1*$^{-/-}$ 48 h DSS distal colon									
	1	2	3	4	5	6	7	8	9	10	1	2	3	4	5	6	7	8	9	10
Cell type score neutrophils	0	0	0	0	0	2	0	0	0	0	0	0	0	0	0	0	0	0	0	0
Cell type score macrophages	0	0	1	0	0	0	0	0	0	0	0	0	0	0	0	0	0	0	0	0
Cell type score lymphocytes	0	0	0	0	0	0	0	0	0	0	0	0	0	0	0	0	0	1	0	0
Erosion/ulceration - depth	0	0	0	0	0	0	0	0	0	0	0	0	0	0	0	0	0	0	0	0
Erosion/ulceration - extent	0	0	0	0	0	0	0	0	0	0	0	0	0	0	0	0	0	0	0	0
Immune cell infiltration - presence	0	0	0	0	0	0	0	0	0	0	0	0	0	0	0	0	0	0	0	0
Immune cell infiltration - extent	0	0	0	0	0	0	0	0	0	0	0	0	0	0	0	0	0	0	0	0
Regeneration	0	0	0	0	0	0	0	0	0	0	0	0	0	0	0	0	0	0	0	0

	WT 48 h DSS proximal colon										WT 48 h DSS distal colon									
	1	2	3	4	5	6	7	8	9	10	1	2	3	4	5	6	7	8	9	10
Cell type score neutrophils	0	0	0	0	0	0	0	0	0	0	0	0	0	0	0	0	0	1	0	0
Cell type score macrophages	0	0	0	0	0	0	0	0	0	0	0	0	0	0	0	0	0	0	0	0
Cell type score lymphocytes	0	0	1	2	0	0	0	0	0	0	2	0	0	0	0	0	0	1	0	0
Erosion/ulceration - depth	0	0	0	0	0	0	0	0	0	0	0	0	0	0	0	0	0	0	0	0
Erosion/ulceration - extent	0	0	0	0	0	0	0	0	0	0	0	0	0	0	0	0	0	0	0	0
Immune cell infiltration - presence	0	0	0	0	0	0	0	0	0	0	0	0	0	0	0	0	0	0	0	0
Immune cell infiltration - extent	0	0	0	0	0	0	0	0	0	0	0	0	0	0	0	0	0	0	0	0
Regeneration	0	0	0	0	0	0	0	0	0	0	0	0	0	0	0	0	0	0	0	0
